# Data supporting ER stress response in NG108-15 cells involves upregulation of syntaxin 5 expression and reduced amyloid β peptide secretion

**DOI:** 10.1016/j.dib.2015.10.025

**Published:** 2015-11-07

**Authors:** Kei Suga, Ayako Saito, Kimio Akagawa

**Affiliations:** Department of Cell Physiology, Kyorin University School of Medicine, Mitaka, Tokyo 181-8611, Japan

## Abstract

This data contains insights into the upregulation of the ER-Golgi-soluble *N*-ethylmaleimide-sensitive factor-attachment protein receptors (ER–Golgi SNAREs) syntaxin 5 (Syx5) by ER stress and the downregulation of Syx5 by apoptosis induction. Use of the protein synthesis inhibitor verified the *de novo* synthesis of Syx5 under ER stress in NG108-15 cells. We also provide validation data for the increase of Syx5 expression caused by ER stress using different chemical compound and overexpression analysis. Interpretation of our data and further extensive insights into the role of Syx5 in βAPP processing under ER stress can be found in Suga et al. (2015)[1].

**Specifications Table**

**Table:** 

Subject area	*Biology*
More specific subject area	*Apoptosis, ER Stress response, ER–Golgi SNAREs, syntaxin*
Type of data	*Text file, graph, figure*
How data was acquired	*Western blotting, Calcium imaging, microscopy, time lapse imaging*
Data format	*Analyzed*
Experimental factors	*NG108*-*15 cells were treated with apoptosis inducer or different ER stress inducer.*
Experimental features	*ER stress or apoptosis induced cells were processed for western blotting, and calcium imaging. Western blotting was also performed with cells overexpressed with Syx5 isoforms.*
Data source location	*Kyorin University School of Medicine, Tokyo, Japan*
Data accessibility	*All data are provided in this article*

**Value of the data**

• Data show the effect of apoptosis induction on the amount of ER–Golgi SNARE Syx5 in NG108-15 cells.
• Data show the effect of different ER stress inducers on the expression of ER–Golgi SNAREs Syx5 and Bet1.
• Effect of Syx5 overexpression on the expression of BiP/GRP78 protein was assessed.

## 1 Data, experimental design, materials and methods

**Fig. 1:**
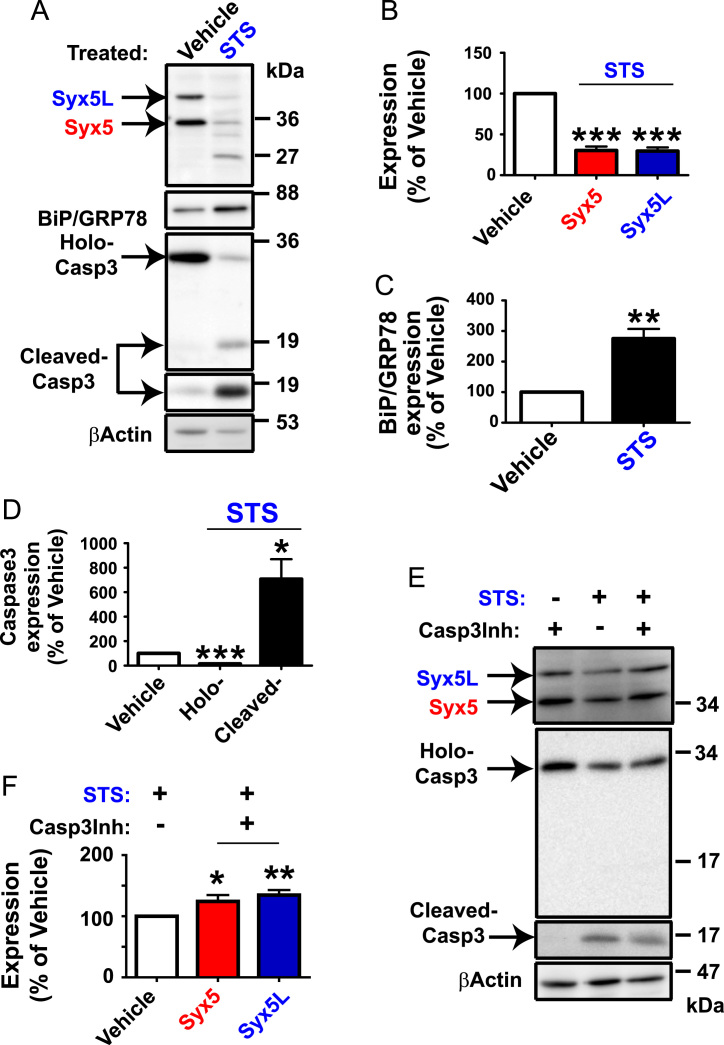
Syx5 expression is decreased by Staurosporine-induced apoptosis in NG108-15 cells. NG108-15 cells were treated with 0.1 μM Staurosporine (STS), a potent inducer of apoptosis for 16 h. A. Total cell extracts were subjected to Western blotting analyses with the antibodies for Syx5 (mouse monoclonal 1C5), for holo- and cleaved forms of Caspase3 (mouse monoclonal CPP32), and for cleaved form of Caspase3 (rabbit monoclonal 5A1). Representative image from 6 independent samples is shown. β-Actin antibody was used to verify that equal amounts of protein were loaded in each lane. Note that two Syx5 isoforms; 35-kDa isoform (designated Syx5), the 42-kDa isoform (designated Syx5L) are both decreased by the STS treatment (A and B). STS treatment significantly reduced the intracellular level of Syx5 isoforms to approximately 30% of the vehicle treated cells (*t*-test, ***P<0.001) while BiP/GRP78 was upregulated to approximately 275% of the vehicle treated cells (*t*-test, **P<0.01). The 27-kDa fragment may be the N-terminal fragment of Syx5, because the antibody 1C5 recognizes N-terminus of Syx5 protein [3] and the cleavage site in Syx5 due to activated/cleaved Caspase3 has been reported to be mapped to amino acid residues 206–209 of Syx5 protein. C. STS elevates the expression of ER stress sensor protein BiP/GRP78. D. Caspase3 was activated by the STS treatment, since its holoprotein was decreased to 14.4%, and its activated form (cleaved Caspase3) showed 7-fold increase compared to the vehicle treated cells (*t*-test, *P<0.05, ***P<0.001). E. NG108-15 cells were treated simultaneously for 16 h with cell permeable Caspase 3 inhibitor (5 μM) together with or without STS. The cell extracts were prepared and subjected to WB. Representative image is shown. Although the cleaved form of Caspase3 was not detectable with antibody CPP32, it was detectable with the use of 5A1 antibody which is specific for cleaved form of Caspase3. F. Expression level of Syx5 proteins that were normalized with that of the β-Actin expression was quantified. Syx5 proteins are actually degraded by Caspase3 in NG108-15 cells, since the degradation of both Syx5 isoforms were suppressed by the treatment with Caspase3 inhibitor in cell treated with STS (*t*-test, *P<0.05, **P<0.01).

**Fig. 2:**
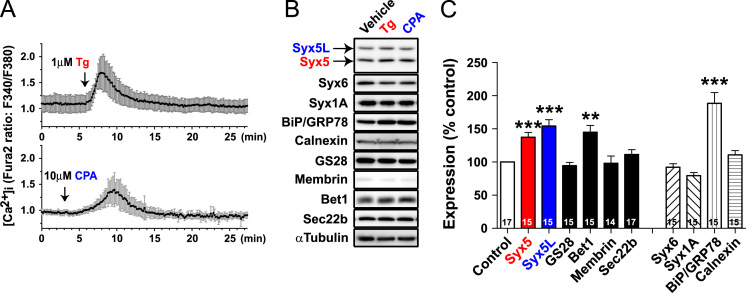
Effect of cyclopiazonic acid on the expression of SNARE proteins. NG108-15 cells were treated with the SERCA inhibitor cyclopiazonic acid (CPA), and Western blotting was performed as described in Section 1. CPA is a widely used chemically synthesized compound for treating various cells. A. NG108-15 cells were treated with 5 μM Fura2-AM (AM; acetoxy-methyl ester, Molecular Probes) for 25 min, washed, and allowed to stand for 15 min in normal medium. After changing the medium to HEPES containing Hank׳s balanced salts solution, the intracellular Ca^2+^ concentration ([Ca^2+^]_i_) was measured. Fura2 ratio imaging analysis was performed using an ARGUS50 Ca^2+^ imaging system (Hamamatsu Photonics, Hamamatsu, Japan) and an inverted microscope (Daiphot300, Nikon, Japan) equipped with a CCD camera (C2400-80, Hamamatsu Photonics). Fluorescent images were taken at excitation wavelengths of 340 and 380 nm and were acquired every 10 s. Cells were treated with 1 μM Tg or 10 μM CPA at the time shown by arrows. The ratio of Fura2 fluorescence (F340 nm/F380 nm) was calculated and plotted against time, and the values from single cells were averaged at each time point and expressed as the mean±SD (*n*=10 for Tg and *n*=7 for CPA). Both SERCA inhibitors induced a transient increase in [Ca^2+^]_i_. B. Extracts from the cells treated with CPA or Tg for 16 h were examined by Western blotting using the indicated antibodies. C. Expression level of Syx5 proteins that were normalized with that of the α-tubulin expression was quantified. Summary of the quantification of various protein expressions in cells treated with CPA. The number of samples examined is indicated in an inset within each column. Similar to the case with Tg, CPA treatment did not induce upregulation of Syx1A and Syx6 expression (79.5±4.6% and 92.2±5.3% of the control, respectively). Although the level of GS28 and membrin expression (94.5±5.0% and 98.0±11.1% of the control, respectively) did not change, that of Syx5, Syx5L, and Bet1 (137.4±7.0%, 154.3±9.7%, and 144.9±10.2% of the control, respectively) was markedly upregulated following CPA treatment. **P< 0.01 and ***P< 0.001 *vs.* control or vehicle, as determined by a *t*-test.

**Fig. 3:**
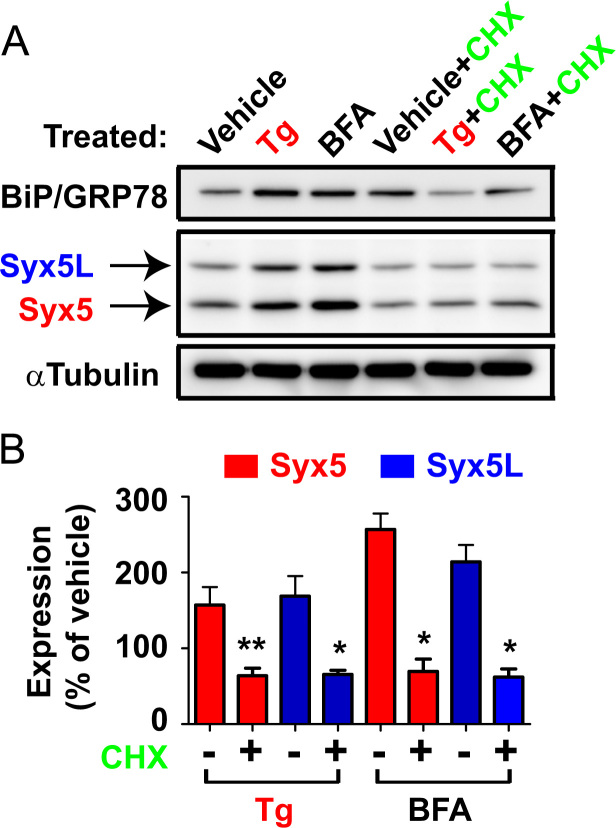
Upregulation of Syx5 isoforms is due to *de novo* synthesis of Syx5 proteins. NG108-15 cells were treated with Tg (1 μM) or BFA (2 μg/mL) for 16 h in the presence or absence of the protein synthesis inhibitor cyloheximide (CHX, 10 μg/mL) and subjected to Western blotting. A. Representative image from 4 independent samples is shown. In the presence of CHX, cells treated with Tg or BFA did not exhibit upregulation of Syx5 isoforms as well as positive ER stress marker protein BiP/GRP78. B. Quantification of the expression level of Syx5 protein in A. *P<0.05, **P<0.01, *vs.* vehicle by a *t*-test.

**Fig. 4:**
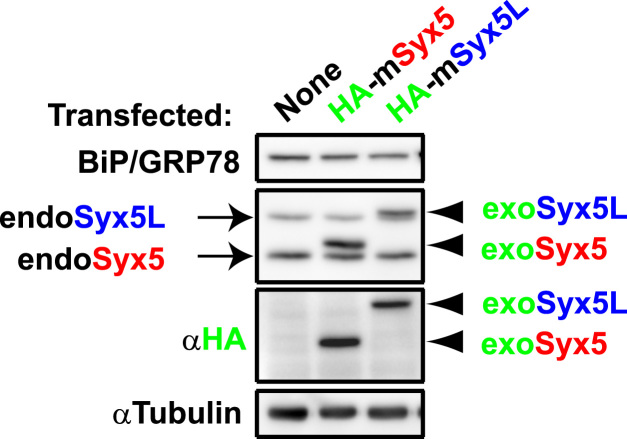
Overexpression of either of the Syx5 isoforms does not induce BiP/GRP78 expression. NG108-15 cells were transfected with expression plasmids for the cDNAs encoding HA-tagged full-length mouse Syx5 (mSyx5-pcDNA3HAN) and Syx5L (mSyx5L-pcDNA3HAN), and Western blotting was performed. A representative image is shown. Under conditions in which each of the Syx5 isoforms was overexpressed, BiP/GRP78 expression was not upregulated.

Syx5 is a member of ER–Golgi SNAREs and is a major player in the transport processes in the early secretory compartment [2]. Moreover, we previously showed that manipulation of the ER–Golgi SNARE Syx5 causes changes in Golgi morphology [3] and the processing of Alzheimer׳s disease (AD)-related proteins [4], [5], [6]. Here, we used western blotting analyses to validate upregulation of Syx5 proteins by ER stress in NG108-15 cells. We confirmed the effect of stress-inducing reagents on intracellular calcium concentration using Ca^2+^ imaging technique (Fig. 2). We also showed that Syx5 is significantly downregulated by Caspase 3 under staurosporine-induced apoptosis (Fig. 1). In addition, we assessed the effect of another ER stress inducer cyclopiazonic acid (CPA) which is a chemical compound on Syx5 expression in NG108-15 cells (Fig. 2). *De novo* synthesis of Syx5 isoforms under ER stress was verified using protein synthesis inhibitor (Fig. 3). Finally, we verified the lack of BiP/GRP78 induction by overexpression of Syx5 isoforms (Fig. 4).

### 1.1 Materials

Hoechst 33342, and brefeldin A (BFA) were purchased from Sigma Chemical Co. (St. Louis, MO, USA). BFA was stored as a 5 mg/mL solution in methanol. Staurosporine (STS), thapsigargin (Tg), and cyclopiazonic acid (CPA) were purchased from Merck (Darmstadt, Germany) and dissolved in dimethyl sulfoxide (DMSO). Cyloheximide (CHX) and cell permeable Caspase3 inhibitor (II: Z-D(OMe)E(OMe)VD(OMe)-FMK) were obtained from Merck. A protease inhibitor cocktail was purchased from Wako Chemicals (Osaka, Japan). All other reagents were of the highest grade available, unless otherwise noted.

### 1.2 Antibodies

Mouse anti-Syx5 monoclonal antibody (clone 1C5) was prepared as described previously [3]. Epitope mapping showed that the antibody 1C5 recognizes N-terminal 1–66 amino acid residues of Syx5 [3]. Mouse monoclonal antibodies against α-tubulin, and Sec22b, rabbit polyclonal antibody against β-actin were obtained from Sigma. Mouse monoclonal antibodies against caspase 3/CPP32, BiP/GRP78, and Syx6 were obtained from BD Transduction Laboratories (San Diego, CA, USA). Antibodies against GS28 and Bet1 were obtained from Stressgen (Victoria, BC, Canada). Anti-calnexin and -membrin antibodies were obtained from Stressgen. Rat monoclonal anti-hemagglutinin (HA) antibody 3F10 was purchased from Roche Diagnostics (Indianapolis, IN, USA). Rabbit monoclonal antibody (5A1) against cleaved caspase-3 (Asp175) was obtained from Cell Signaling Technology (Danvers, MA, USA).

### 1.3 Cell culture and transfection

Mouse neuroblastoma and rat glioma hybrid NG108-15 cells were cultured in Dulbecco׳s modified Eagle׳s medium containing 4 mM l-glutamine, 100 U/mL of penicillin, 100 µg/mL of streptomycin, 2.5 mM hypoxanthine, 10 µM aminopterin, 0.4 mM thymidine, and 10% fetal bovine serum in a humidified incubator with 5% CO_2_ at 37 °C, as described previously [6]. The cells were inoculated into type IV collagen-coated 6- or 24-well plates or 100-mm dishes (BD Bioscience, Bedford, MA, USA) for extract preparation, into poly-l-lysine-coated 35-mm dishes (BD Bioscience) with glass bottoms for time lapse imaging analyses. The cells were transfected with plasmids using FuGENE6 transfection reagent (Promega, Madison, WI, USA).

### 1.4 Extract preparation, SDS-PAGE, western blotting

After treatment, cells were washed three times with 4 mL of phosphate-buffered saline (PBS; pH 7.4), harvested with a cell scraper, and recovered by centrifugation at 1500*g* at 4 °C for 3 min. The resultant cell pellets were lysed in 150 μL of extraction buffer (50 mM Tris–HCl [pH 7.5], 0.15 M NaCl, 1% Triton X-100) containing a protease inhibitor cocktail and sonicated for 30 s. Insoluble material was removed by centrifugation at 15,000*g* for 10 min, and the resultant supernatant fraction was used as the extract sample. After determination of the protein content by the Bradford method [7] using bovine serum albumin as a standard, Laemmli sample buffer [8] was added for use in Tris/glycine SDS-PAGE. For Tris/tricine SDS-PAGE, Serva blue G (SBG; Serva Electrophoresis, Heidelberg, Germany) sample buffer (50 mM Tris–HCl [pH 6.8], 4% SDS, 12% glycerol, 2% 2-mercaptoethanol, and 0.01% SBG) was used [9]. After solubilization in SDS-containing sample buffer for 30 min at 37 °C, the extract samples were frozen and stored at −80 °C until analysis. Samples were subjected to Tris/glycine 10 or 12% SDS-PAGE or Tris/tricine 16.5% SDS-PAGE and Western blotting as described previously [1], [3], [5], [6], [10], with minor modifications. Immunoreactive bands were visualized using enhanced chemiluminescence (ECL) reagents (GE Healthcare, Piscataway, NJ, USA) or Pierce Western blotting substrate (Thermo Scientific) and visualized using an LAS3000 imaging system (FujiFilm, Tokyo, Japan). The immunoblots were also exposed to Hyperfilm (GE Healthcare).

### 1.5 Plasmid construction

Expression plasmids for the cDNAs encoding HA-tagged full-length mouse Syx5 (mSyx5-pcDNA3HAN) and the long isoform of Syx5 (Syx5L; mSyx5L-pcDNA3HAN) were cloned from C57black strain mice using conventional RT-PCR methods. Cloning of the cDNAs encoding Syx5L was performed by generating oligonucleotide primers based on the sequence of mRNA transcript variant 1 (GenBank accession number: NM_019829). The cDNA fragments encoding full-length mSyx5 and mSyx5L were produced by PCR using a 5′-primer containing an additional in-frame *BamH*I site, and the 3′-primer was constructed by inserting PCR fragments digested with *BamH*I and *Not*I into the *BamH*I–*Not*I site of the pcDNA3-HAN vector described previously. Sequences of all constructs were verified both by direct sequencing on an ABI373A Sequencer with a BigDye filter or on an ABI3730 Sequencer (Life Technologies Japan, Tokyo, Japan) and by analysis with the appropriate restriction enzymes.

### 1.6 Ca^2+^ imaging

Cells were treated with 5 μM Fura2-AM (AM; acetoxy-methyl ester, Molecular Probes) for 25 min, washed, and allowed to stand for 15 min in normal medium. After changing the medium to HEPES containing Hank׳s balanced salts solution, the intracellular Ca^2+^ concentration ([Ca^2+^]_i_) was measured as described previously [11]. Fura2 ratio imaging analysis was performed using an ARGUS50 Ca^2+^ imaging system (Hamamatsu Photonics, Hamamatsu, Japan) and an inverted microscope (Daiphot300, Nikon, Japan) equipped with a CCD camera (C2400-80, Hamamatsu Photonics). Fluorescent images were taken at excitation wavelengths of 340 and 380 nm and were acquired every 10 s.

### 1.7 Data analysis and statistics

The enhanced chemiluminescence signals from western blot analyses and the amount of each PCR product were analyzed using MultiGauge software (FujiFilm, Tokyo, Japan). Statistical analyses were performed using GraphPad Prism software (GraphPad Software, La Jolla, CA). Data are presented as mean±SEM, with ‘*n*’ indicating the number of samples examined. A *t*-test was used to determine the statistical significance of differences between values.
